# Gut Microbes Secretions May Trigger Mononuclear Cell Migration and Offer Comorbidity Mechanism Between Inflammatory Bowel Disease and Diabetic Retinopathy

**DOI:** 10.1155/mi/9894696

**Published:** 2025-11-24

**Authors:** Bingyu Xu, Shuduan Wu, Junfei Huang, Mengqing Lin, Qiang Liu, Xiyun Li, Min Fu, Zhaoxia Xia, Guoguo Yi

**Affiliations:** ^1^The Second Clinical School, Southern Medical University, Guangzhou, Guangdong, China; ^2^Department of Ophthalmology, The Sixth Affiliated Hospital, Sun Yat-sen University, Guangzhou, Guangdong, China; ^3^Biomedical Innovation Center, The Sixth Affiliated Hospital, Sun Yat-sen University, Guangzhou, China; ^4^The First Clinical School, Southern Medical University, Guangzhou, Guangdong, China; ^5^Department of Ophthalmology, Zhujiang Hospital of Southern Medical University, Guangzhou, Guangdong, China

**Keywords:** bile acids, blood-retinal barrier, diabetic retinopathy, gut microbiota, inflammatory bowel disease, L-kynurenine, metabolite, peripheral blood mononuclear cells

## Abstract

**Background:**

Researches have found that the gut microbiota stimulate inflammation by releasing proinflammatory chemicals, including bile acids (BAs) and L-kynurenine. In the present study, our purpose is to investigate the potential functional roles of gut microbes and their secretions in the comorbidity mechanism between inflammatory bowel disease (IBD) and diabetic retinopathy (DR).

**Methods:**

Specific existing gut microbes among IBD and diabetes mellitus (DM) patients were gained and analyzed to figure out the proinflammatory chemicals secreted by specific existent microbes. The expression data of peripheral blood mononuclear cells (PBMCs) were obtained and underwent differentially expressed genes (DEGs) analysis and weighted gene co-expression network analysis (WGCNA) to find out potential effects of microbes secretions on PBMCs. Single-cell analysis was conducted to elucidate the underlying cytological comorbidity mechanisms.

**Results:**

The results of the study showed that the secretions of abnormal gut microbes in IBD and DM patients, such as BAs and L-Kynurenine, can stimulate abnormal immune response. PBMCs can be activated, mobilized, and disrupt vascular endothelial barriers upon exposure to proinflammatory substances generated by *Veillonella* and *Clostridium*, the dysbiotic gut microbiota in IBD, which exacerbate retinal inflammation and worsening DR pathological conditions. Furthermore, genes related to PBMC migration, such as RSU1, FN1, and CD9, are activated in PBMCs after exposure to pro-inflammatory chemicals from abnormal gut microbes, offering a genetic comorbidity mechanism for IBD and DR. We also unraveled that IBD can promote the proliferation and activation of effector memory T (Tem) cells, which also showed elevation in DR patients, thereby providing a cellular basis for the comorbidity between IBD and DR.

**Conclusion:**

The aberrant gut bacteria in IBD patients might secrete proinflammatory substances, such as L-kynurenine and BAs, which may activate mononuclear cells, especially the Tem. The mononuclear cells subsequently migrated into the retina, exacerbating the situation of DR. This finding underscores the potential of comorbidity mechanism for better treatment of DR in the future.

## 1. Introduction

Inflammatory bowel disease (IBD) is a chronic inflammatory illness of the gastrointestinal (GI) tract, consisting of two idiopathic disorders: Crohn's disease (CD) and ulcerative colitis (UC) [1]. Research indicates that both CD and UC are associated with aberrant pathological changes in gut microbiota, suggesting that gut bacteria may drive the pathological course of IBD [2]. These aberrant bacteria exacerbate inflammatory conditions by generating proinflammatory chemicals [3], such as bile acids (BAs) [4] and L-kynurenine [5]. These chemicals stimulate lymphocytes and monocytes, exacerbating both IBD and inflammatory conditions.

Peripheral blood mononuclear cells (PBMCs) include lymphocytes and monocytes found in peripheral blood. It can be triggered and traversed by metabolites of proinflammatory microorganisms, subsequently transported to organs via the circulatory system [6]. Prior studies revealed that individuals with untreated IBD exhibited significantly elevated levels of DR3 + PBMCs, suggesting that PBMCs in IBD patients are activated [7]. The activated PBMCs, particularly innate lymphoid cells 3 (ILC3) [8] and monocyte-derived dendritic cells (moDCs), augment migratory capacity, resulting in systemic inflammation throughout the body [9].

Diabetic retinopathy (DR) is a recently identified illness associated with PBMCs issues, demonstrated to result from PBMCs dysfunction [10]. DR is a microvascular condition that causes diabetic macular edema (DME), neovascularization, and microaneurysms, potentially culminating in visual impairment and blindness [11]. Retinal inflammation is considered a key factor in the pathological progression of DR [12]. The blood-retina barrier (BRB) effectively prevents the infiltration of immune cells into the retina in healthy individuals [13]. Studies indicate that the formation of focal adhesions can facilitate both the disruption of the BRB and the relocation of PBMCs into the retina, greatly exacerbating DR [14, 15]. Conversely, safeguarding the BRB and preventing the migration of PBMCs might ameliorate this disease. The migration of PBMCs and the start of DR are closely associated with the dysfunction of gut microbiota, particularly in the context of IBD. Liu et al. [16] employed Mendelian Randomization to validate the association between alterations in gut microbiota and DR, suggesting the presence of a “gut-retina” axis. He et al. [17] discovered that gut-homing β7^+^CD4^+^ T lymphocytes in peripheral blood may penetrate the retina via MAdCAM-1, leading to destruction of retinal ganglion lymphocytes (RGCs). IBD demonstrates substantial alterations in gut microbiota, potentially influencing the migration of gut-homing PBMCs to the retina, hence exacerbating DR and suggesting a possible comorbid link between IBD and DR.

T cells, a critical subset of lymphocytes that play a central role in the adaptive immune response, are highly related to the pathological process of both IBD and DR. Xue Bai et al. demonstrated that T follicular helper (TfH) cells facilitate the aggregation of mature DCs within colonic lymphoid follicles and subsequently undergo transdifferentiation into long-lived pathogenic TH1 cells, thereby driving the progression of colitis [18]. Moreover, this study also discovered that T cell subsets, such as TH1 and TH17, are critically involved in the pathogenesis of IBD. Llorián-Salvador et al. [19] illumined that a sub-population of T cells, the regulatory T cells (Treg), plays an important role in the early stage of DR, protecting the neurons from degenerating. All these researches indicated that certain subsets of T cells may contribute to the comorbidity mechanism between IBD and DR.

**Figure 1:**
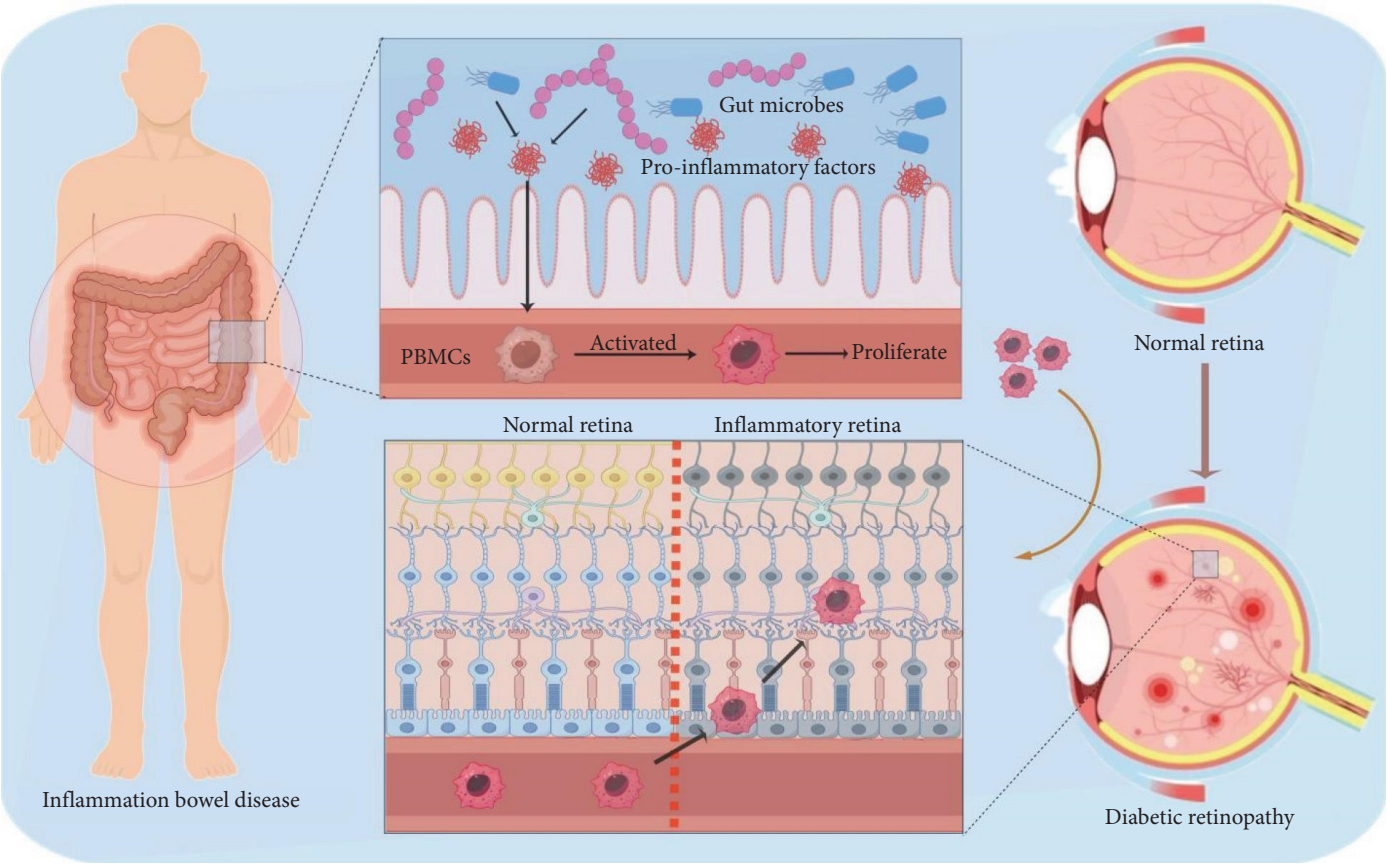
The schematic diagram of mechanism. Aberrant gut bacteria in IBD patients may secrete proinflammatory substances like L-kynurenine and BAs, activating T-cell subsets. Subsequently, these cells migrate into the retina, worsening the retinal inflammatory condition of DR.

We hypothesize that aberrant gut bacteria in IBD patients might secrete proinflammatory substances, such as L-kynurenine and BAs, which may activate mononuclear cells, especially certain subsets of T cells. The mononuclear cells subsequently migrated into the retina, exacerbating the situation of DR (Figure 1). This study collects and analyzes various gut microbiome and gene expression data to elucidate the association between IBD and DR, providing insights into the gut-retina axis and contributing to future treatments for IBD and DR.

## 2. Methods

### 2.1. PBMCs Samples Inclusion and Data Acquisition

A gene expression profile (GSE3365) for IBD was obtained from the GEO database (https://www.ncbi.nlm.nih.gov/geo/). GSE3365 compares the transcriptional patterns of PBMCs between healthy individuals and patients with IBD, comprising 42 healthy participants, 59 patients with CD, and 25 patients with UC. The gene expression of the samples was analyzed using the Affymetrix Human Genome U133A Array (GPL96).

For DR, a gene expression profile (GSE185011) detailing mRNA and lncRNA expression in PBMCs of individuals with diabetes mellitus (DM), DR, diabetic peripheral neuropathy (DPN), and diabetic nephropathy (DN). This study utilized gene expression data from five healthy individuals and five patients with DR. Gene expression is assessed using high-throughput sequencing (GPL24676).

### 2.2. GEO Data Analysis

All data obtained from the GEO database were analyzed and standardized utilizing the R programing language. The differentially expressed genes (DEGs) were examined using the “limma” program. Corrected *p* < 0.05 and |log FC (fold change)| > 1 are utilized as the selection criterion. All data were standardized using log_2_-transformed and incremented by 1.

### 2.3. Gut Microbes Data Collection and Analysis

Data on gut bacteria and analyses of various gut microbial data are available at the Data Repository For Human Gut Microbiota (GMrepo, https://gmrepo.humangut.info) [20]. Conducting a search on GMrepo for gut microbiota in healthy persons and those with Type 2 DM (T2D; PRJNA422434), CD and UC (PRJNA400072), and IBD (PRJNA385949). Following the successful collection of gut microbiome data, GMrepo conducted several analyses of the gut bacteria. The findings were presented as a bar chart.

### 2.4. Functional Analysis and Gene Set Enrichment Analysis

The “clusterProfiler” package in R was utilized for Gene Ontology (GO) and Kyoto Encyclopedia of Genes and Genomes (KEGG) analysis of the candidate genes. Substantial differences were established as an adjusted *p*-value <0.05. The figures are rendered with the “ggplot2” software.

### 2.5. Weighted Gene Coexpression Network Analysis (WGCNA)

WGCNA is a technique for elucidating gene coexpression patterns across various situations to uncover physiologically significant gene networks. The R package “WGCNA” was utilized to construct the gene coexpression network. Initially, the expression matrix was imported into R program. The expression matrix was subsequently cleaned and formatted. A scale-free network was subsequently constructed to compute and determine a soft threshold value for each illness. Gene coexpression modules for each illness were connected with diseases (DR and IBD) following their definition. The genes from the most relevant module were retrieved and further analyzed using a Venn diagram and enrichment analysis.

### 2.6. Single-Cell Analysis

We performed a comprehensive analysis of single-cell RNA sequencing data derived from PBMCs obtained from healthy individuals (GSE255566), patients with IBD (GSE214695), and patients with DR (GSE248284). In this study, the bioinformatics analyses were conducted utilizing the “Seurat” and “SingleR” packages. Cell clustering and sub-population identification were visualized via t-distributed Stochastic Neighbor Embedding (t-SNE) and were automatically annotated using the “SingleR” package.

## 3. Results

### 3.1. Shared Gut Microbes Among IBD and DM Patients Can Stimulate Mononuclear Cells and Secrete Proinflammatory Factors

**Figure 2:**
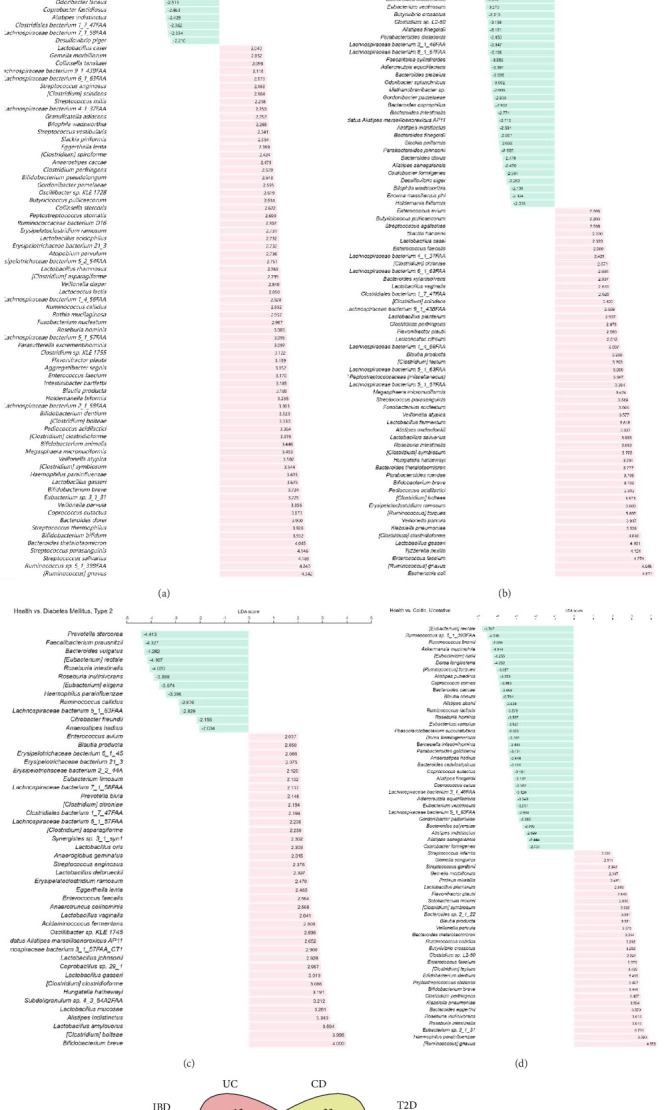
Differential gut microbes analysis. (A) Differential gut microbes of Inflammatory Bowel Disease. (B) Differential gut microbes of Crohn's disease. (C) Differential gut microbes of diabetic mellitus type 2. (D) Differential gut microbes of ulcerative colitis. (E) Venn diagram of the shared differential gut microbes.

To examine the co-occurrence of gut microorganisms in patients with IBD and DM, we analyzed the gut microbial composition in the GMrepo. The distinct gut microbial components of T2D, CD, UC, and IBD were shown using bar plots (Figure 2A–D). The gut microbiota data for IBD, UC, and CD were derived from patients in the United States, whereas the data for T2D were obtained from patients in China. Despite the differences in gut microbiota composition between Asia and North America due to ethnic and dietary variations, several studies have indicated that the gut microbiota profiles of Chinese and American patients with T2D are remarkably similar, including *Clostridium* [21, 22], *Veillonella* [23], *Ruminococcus gnavus* [24], *Streptococcus* [22], and so on. Therefore, it is feasible to conduct comparative studies using gut microbiota data from Chinese T2D patients and those from North America. Conjoint differential gut microorganisms among these groups were collected and analyzed. *Blautia producta*, *Enterococcus*, *Bifidobacterium breve*, *Clostridium*, and *Veillonella* are markedly prevalent in people with T2D and IBD (Figure 2E).

*Veillonella* and *Clostridium* are noted for their capacity to secrete proinflammatory chemicals, such as L-kynurenine [5] and Bas [25]. *Veillonella* are identified as significantly multiplied microbes in GI tracts among America patients with T2D [26]. Moreover, research demonstrated that in IBD models, the abundance of *Veillonella* is significantly elevated and positive related to L-kynurenine level in serum [27, 28]. Furthermore, the serum L-kynurenine concentrations are markedly elevated in individuals with T2D in the United States [29]. L-kynurenine is a metabolite derived from tryptophan. Research indicates that L-kynurenine exacerbates the pathogenic state of CD by activating monocytes and promoting creeping fat accumulation. The upregulation of IDO1 in monocytes catalyzes the production of L-kynurenine, which binds to AHR on the membrane of adipocytes and facilitates lipid mobilization [5]. *Clostridium* is highly related to the secretion of BAs. BAs can induce T cell migration, activation, and differentiation [30]. The aforementioned studies indicate that gut bacteria in the intestines of IBD patients, particularly *Clostridium* and *Veillonella* secrete proinflammatory substances, such as L-kynurenine and BAs, which stimulate mononuclear cells, leading to the migration and activation of PBMCs.

### 3.2. Similar Genes in PBMCs Among IBD and DR Were Activated to Promote Mononuclear Cells Migration and BRB Disruption

**Figure 3:**
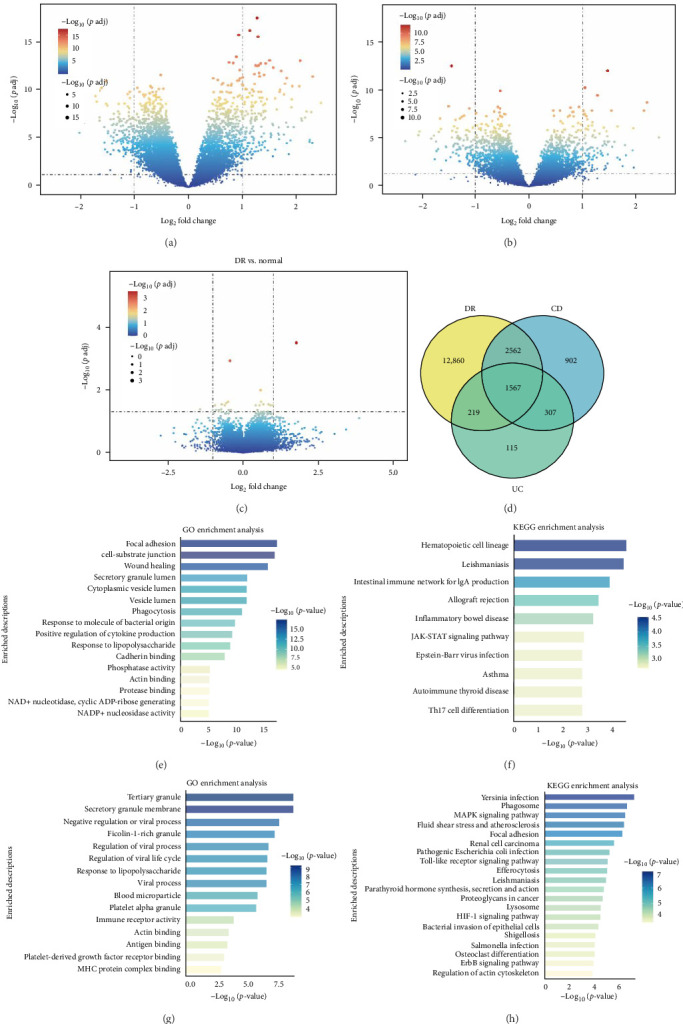
Differentially expressed genes and enrichment analysis. (A–C) DEG results of diabetic retinopathy, ulcerative colitis, and Crohn's disease, respectively. (D) Venn diagram of the DEG results. (E, H) GO enrichment analysis and KEGG pathway analysis of the shared upregulated genes among DR and CD. (F, G) GO enrichment analysis and KEGG pathway analysis of the shared downregulated genes among DR and UC.

Subsequently, we assessed the uniformity of gene expression in PBMCs from IBD and DR patients. DEGs in PBMCs of IBD (GSE3365) and DR (GSE185011) patients were analyzed (Figure 3A–C). In the DR PBMCs sample, 12,860 genes were particularly expressed, whereas 5672 genes were expressed in the IBD PBMCs sample, 2208 in CD, and 5538 in UC, respectively.

The DEGs in PBMCs from patients with CD and UC were compared to those from patients with DR (Figure 3D). A total of 1567 DEGs underwent GO functional enrichment analysis and KEGG pathway enrichment analysis (Figure 3E–H). The common upregulated genes associated with DR and CD primarily pertain to “focal adhesion,” “cell-substrate junction,” “phagocytosis,” “response to bacterial molecules,” and “positive regulation of cytokine production,” all of which are closely linked to the migration of mononuclear cells and the disruption of microvascular endothelial junctions. The downregulated genes common to UC and DR focus on “tertiary granule,” which is strongly linked to the transfer of immune cells from the bloodstream into tissues, leading to the breakdown of the microvascular endothelial barrier. KEGG pathway enrichment analysis was conducted. Common genes predominantly focused on “phagosome,” “MAPK signaling pathway,” “focal adhesion,” “retinal cell carcinoma,” “inflammatory bowel disease,” and “Th17 cell differentiation.” All of the above enrichment indicates that the relocation-related genes were simulated in PBMCs from IBD patients, enhancing the chemotaxis ability, catalyzing the undermining of the retinal microvascular endothelial barrier, which is an important component of BRB, and elevating retinal inflammatory level, exacerbating DR condition.

### 3.3. PBMCs in IBD Patients Migrate to Retina and Propelling DR by Breaking Retinal Microvascular Barrier

**Figure 4:**
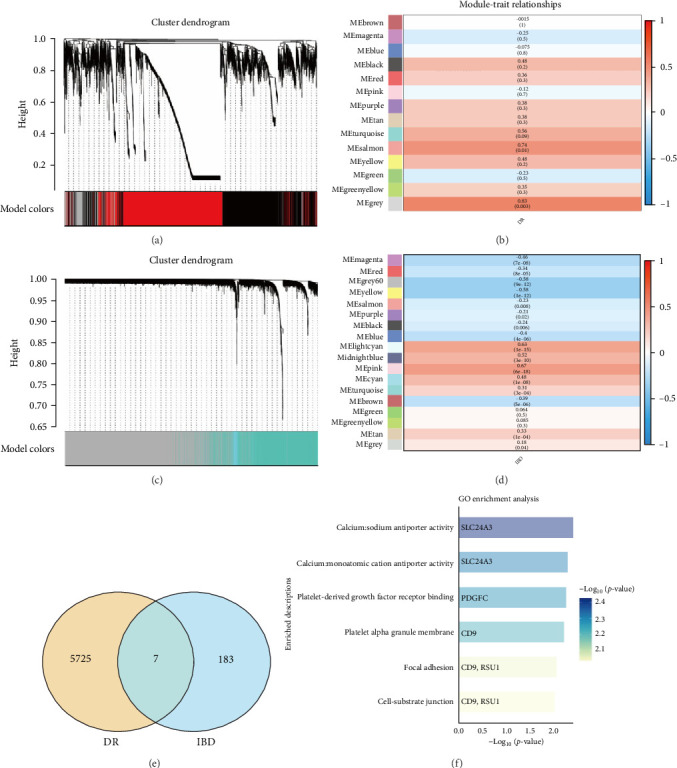
WGCNA and enrichment analysis. (A, B) WGCNA results for DR. (C, D) WGCNA results for IBD. (E) Venn diagram of the most related genes among DR and IBD. (F) GO enrichment analysis of the joint most related genes.

Following DEG analysis, WGCNA was utilized to identify the gene module most correlated with clinical outcomes (Figure 4A–D). Gene expression in IBD (GSE3365) and DR (GSE185011) patients' PBMCs was grouped based on its correlation with clinical outcomes.

To create a DR scale-free network, the soft threshold (*R*^2^ = 0.85) was established at 17 (Figure S1B), whereas IBD was designated at 8 (Figures 4A,C and S1A). The WGCNA identified 14 modules linked to the occurrence of DR and 18 modules linked to the prevalence of IBD. Each module was designated by a distinct hue. The genes in the “gray” module had a substantial positive correlation with DR (gray module: *r* = 0.83, *p*=0.003) (Figure 4B). The “pink” module had a significant positive correlation with IBD (pink module: *r* = 0.67, *p*=6e–18) (Figure 4D).

To analyze the correlation between IBD and DR in PBMCs, the “gray” module from the DR analysis and the “pink” module from the IBD analysis were isolated and subjected to Venn diagram evaluation (Figure 4E). Seven potential genes linked to both DR and IBD were found through the intersection of genes in the aforementioned target modules: CD9, MEST, PDGFC, PLCL1, RSU1, SLC24A3, and TPST2. GO functional analysis was conducted to elucidate the probable mechanism behind the comorbidity of IBD and DR (Figure 4F). Results indicate that PBMCs in both IBD and DR patients influence calcium monoatomic antiporter function, focal adhesion, and cell-substrate junctions. Research has established that the calcium monoatomic antiporter is significantly associated with the degradation of the microvascular endothelial barrier [31, 32], while the expression of genes linked to focal adhesion [33, 34] and cell-substrate junctions [34] in PBMCs correlates with their migration and relocation, further compromising the microvascular barrier. These data suggested that IBD may exacerbate DR by promoting the breakdown of the retinal microvascular endothelial barrier, altering the BRB, and intensifying retinal inflammation, ultimately leading to neuronal degeneration.

### 3.4. The Proliferation and Activation of Tem Provide a Cellular Comorbid Mechanism Between DR and IBD

**Figure 5:**
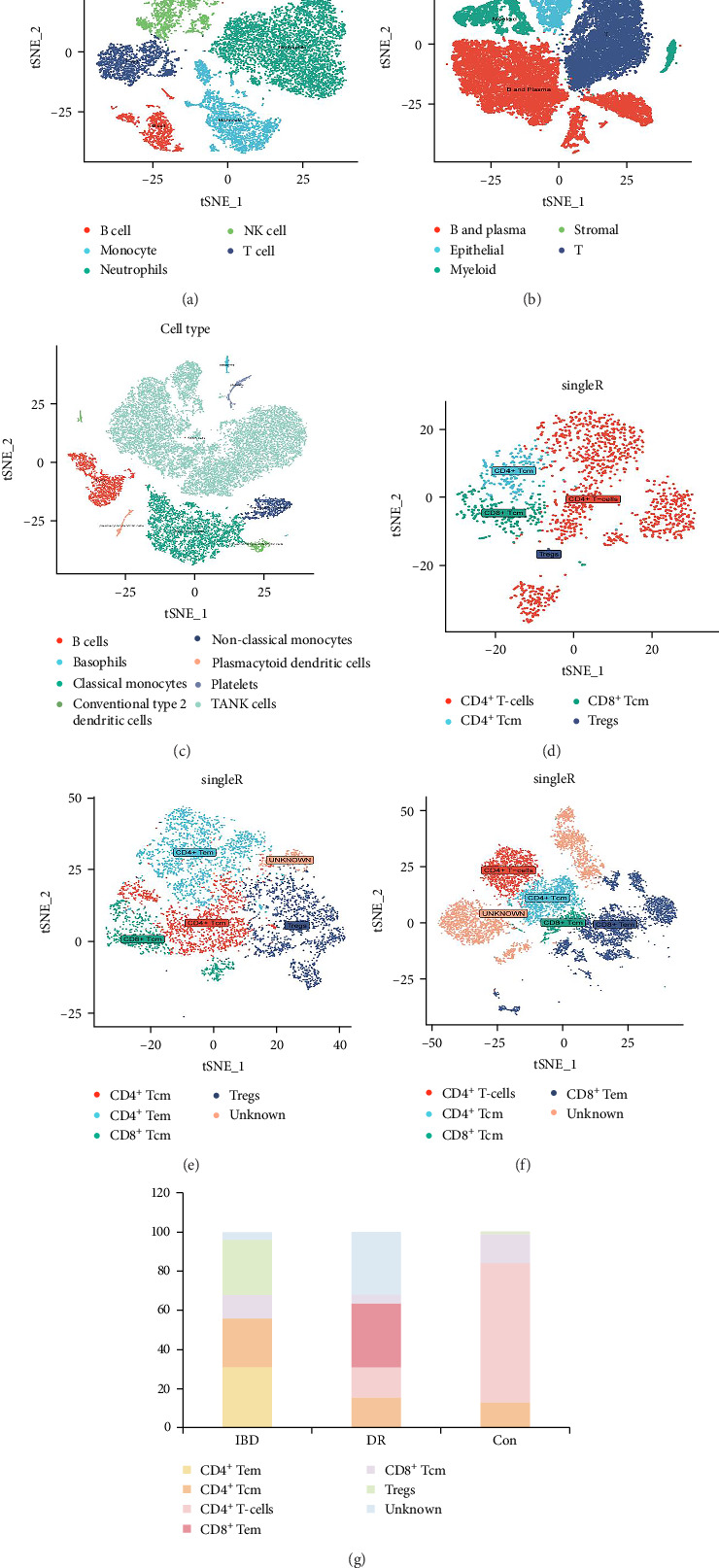
Single-cell Analysis of healthy people, IBD patients and DR patients. (A) Cells clustering of PBMCs among healthy people. (B) Cells clustering of PBMCs among IBD patients. (C) Cells clustering of PBMCs among DR patients. (D) Sub-population analysis of T cells among healthy people. (E) Sub-population analysis of T cells among IBD patients. (F) Sub-population analysis of T cells among DR patients. (G) The proportion of each T cells among IBD patients (IBD), DR patients (DR), and healthy controls (Con).

Following the identification of potential comorbid genes between IBD and DR via WGCNA, we intend to delve further into the cellular-level relationship between IBD and DR. In order to further investigate the cytological etiology between DR and IBD, we exploited single-cell analysis to explore the cellular characteristics of healthy people (GSE255566), IBD patients (GSE214695), and DR patients (GSE248284) (Figure 5A–G).

The cellular constituents of PBMCs cells were investigated using cell clustering analysis. After the conduct of cellular clustering analysis among PBMCs sourced from healthy individuals, patients with IBD, and those with DR, we have reported a statistically significant divergence in the composition of T-cells not only between IBD patients and healthy controls, but also between DR patients and healthy controls (Figure 5). This seminal observation suggests that gut microbiota secretions may play a pivotal role in exacerbating inflammation through the proliferation of certain subsets of T-cells. Consequently, an extensive analysis of cellular sub-populations was undertaken to accurately identify and characterize the T-cell sub-populations that were affected by these alterations. Effector memory T (Tem) cells, characterized as antigen-experienced lymphocytes, reside predominantly in peripheral tissues. In our study, we utilized the SingleR package to identify specific T-cell sub-populations in healthy individuals, patients with IBD and those with DR. The analytical results revealed a significant increase in the ratio of Tem cells in both DR and IBD patient cohorts when compared to healthy controls (Figure 5). Tem cells are highly regarded as indicators of immune system activation due to their cytotoxicity and ability to secrete inflammatory cytokines [35]. Such activation may lead to the disruption of the BRB and ultimately exacerbate retinal inflammation. Our study has identified and confirmed that the secretions from gut microbiota in IBD, such as BAs and L-kynurenine, promote the proliferation and activation of Tem cells, which can exacerbate retinal inflammation in the early occurrence and promote the pathological progression of DR.

## 4. Discussion

This study revealed the distinct gut microbiota common to DR and IBD, revealing their association with PBMCs activation and migration in DR. The movement of PBMCs, especially the Tem cells, crossing over the microvascular endothelial barrier is the most pertinent pathogenic pathway common to DR and IBD, promoted by metabolites from gut bacteria, including L-kynurenine and BAs. We identified a potential comorbidity mechanism linking DR and IBD: L-kynurenine and BAs produced by the abnormal gut microbiota of IBD patients promote DR by enhancing the migration of PBMCs into the retina and compromising the microvascular endothelial barrier, leading to increased retinal inflammation and exacerbating the pathological condition of DR. L-kynurenine and BAs stimulate monocytes and lymphocytes through different mechanisms. L-kynurenine, a significant metabolite of tryptophan, functions as a ligand for the aryl hydrocarbon receptor (AHR) [36], influencing both ILCs and moDCs. The AHR is essential for the formation of ILC3 in the central lymphatic nodes by activating the Runx3-RorγT pathway [37]. ILC3s can facilitate inflammation and chemotaxis by the secretion of IL-17 [38, 39] and by activating CD4^+^ T cells [40]. The differentiation of moDCs is also promoted by L-kynurenine via the induction of BLIMP-1 [41]. BAs are prevalent in the human GI tract, where they are metabolized by bacteria, resulting in the generation of a substantial array of bioactive metabolites. Research indicates that BAs can facilitate the migration of intestinal lymphocytes into the bloodstream by enhancing the production of VCAM-1 and ICAM-1 [42], consequently activating the S1PR2 pathway [34, 43], which leads to the development of adhesion molecules. These adhesion molecules promote the interaction between lymphocytes and vascular endothelial cells, facilitating their migration to other tissues. Furthermore, the aberrant metabolism of BAs might influence monocyte production, resulting in a proinflammatory circulation [3].

Particular genes were activated in PBMCs, leading to the migration of immune cells. RSU1, FN1, and CD9 are distinguished by their capacity to facilitate the movement of PBMCs. These particular expressed genes are strongly associated with clinical outcomes in DR and IBD. The functional enrichment analysis revealed that these genes may facilitate the formation of focal adhesion between PBMCs and vascular endothelial cells, resulting in the migration of PBMCs to the tissue matrix. Molecular mechanisms are found as well. For example, RSU1 expression levels have a favorable correlation with the infiltration of CD4^+^ T cells, macrophages, and DCs [44], facilitating PBMCs movement through interaction with PINCH1 and influencing the actin cytoskeleton of cells [45]. The overexpression of FN1 in macrophages exacerbates inflammatory conditions by secreting TNFα, MCP-1, and THBS-1 [46]. Furthermore, CD9, a crucial gene in cellular adhesion and transport, facilitates cell mobility by stimulating the phosphorylation of focal adhesion kinase (FAK) [47]. The migration of PBMCs ultimately leads to the malfunction of the microvascular endothelial barrier, resulting in disruption of the BRB and retinal inflammation.

The existence of gut-eyes axis had been provided by several researches. For example, Wang et al. [48] reported that the microbial abundance in glaucoma patients undergoes significant alterations, and the activation of the AhR pathway serves to protect retinal ganglion cells, suggesting the existence of the gut-eye axis. Shen et al. [49] discovered that ACHP effectively ameliorates intestinal barrier function and restores gut microbiota homeostasis, thereby reducing retinal inflammation and oxidative stress in DR. These studies collectively underscore the intimate connection between the gut and the retina, thereby suggesting the existence of the gut-eye axis.

The microvascular endothelial barrier constitutes a component of the BRB, inhibiting the infiltration of immune cells into the retina and sustaining a low inflammatory state inside the retina [50]. When the endothelial barrier is compromised, PBMCs may infiltrate the retina, leading to retinal inflammatory lesions, which can subsequently result in neovascularization, neuronal degeneration, and the progression of DR. This study posits that chemicals from gut bacteria would induce the migration and translocation of PBMCs into the retina, compromising the microvascular endothelial barrier and the BRB. Immune cells infiltration into the retina can induce the production of VEGF [51, 52] and HIF-α [53, 54], worsening retinal inflammation and oxidative stress, resulting in neovascularization, neuronal damage, and potential blindness.

## 5. Conclusion

In conclusion, the migration of PBMCs and the breakdown of the microvascular endothelial barrier are implicated in IBD and DR, perhaps explaining the comorbidity between these conditions. Furthermore, the gut microbiota in IBD might induce the migration of PBMCs such as Tem cells via metabolites, particularly L-kynurenine and BAs, hence promoting the pathogenic processes of DR. These findings elucidated the mechanism between IBD and DR and served as a reference for subsequent inquiry.

## 6. Limitations

Our study has certain limitations. First, despite the exclusion of batch effects, all data utilized in this study were sourced from public databases. Second, although all findings in this study have been corroborated by multiple methodologies, such as the identification of DEGs, WGCNA, and single-cell RNA sequencing analysis, the thoroughly analysis for the detailed molecular mechanisms underlying the comorbidity of IBD and DR await further investigation, which need to be validated by further laboratory-based experiments and cohort studies.

## 7. Future Directions and Clinical Implications

We plan to culture and extract secretions from aberrant gut microbiota in IBD patients, such as L-kynurenine and BAs, and verify their stimulatory effects on T-cell subsets and their promotional effects on DR at both the cellular and animal levels in the next phase of our study.

If the findings of this study are further corroborated by subsequent researches, it will provide novel insights into the complications of IBD and the mechanisms by which gut microbiota contribute to these complications. Additionally, it will enhance our understanding of the role of the “gut-eye axis.” This could facilitate the development of therapeutic strategies targeting specific T-cell subsets, thereby alleviating complications associated with IBD.

## Data Availability

The gut microbes datasets analyzed during the current study are available in the Data Repository For Human Gut Microbiota (GMrepo), https://gmrepo.humangut.info/data/project/PRJNA400072, reference number: PRJNA400072, https://gmrepo.humangut.info/data/project/PRJNA385949, reference number: PRJNA385949, and https://gmrepo.humangut.info/data/project/PRJNA422434, reference number: PRJNA422434. The gene expression data that support the findings of this study are available in GEO database at https://www.ncbi.nlm.nih.gov/geo/query/acc.cgi?acc=GSE214695, reference number: GSE214695 and https://www.ncbi.nlm.nih.gov/geo/query/acc.cgi, reference number: GSE248284. The single-cell RNA sequencing data mentioned in this study are available in GEO database at https://www.ncbi.nlm.nih.gov/geo/query/acc.cgi?acc=GSE255566, reference number: GSE255566, https://www.ncbi.nlm.nih.gov/geo/query/acc.cgi, reference number: GSE214695, and https://www.ncbi.nlm.nih.gov/geo/query/acc.cgi, reference number: GSE248284.

## Supporting Information

Supporting Information The Supporting Information are about WGCNA Scale independence and mean connectivity results. Supporting Figure S1. (A) Scale independence and mean connectivity results for IBD. (B) Scale independence and mean connectivity results for DR. The supporting informations have been uploaded.
