# Supplementary material for: Gut Microbes Secretions May Trigger Mononuclear Cell Migration and Offer Comorbidity Mechanism Between Inflammatory Bowel Disease and Diabetic Retinopathy
Source: Mediators Inflamm. 2025 Nov 24;2025:9894696. doi: 10.1155/mi/9894696 (PMC12668851; doi:10.1155/mi/9894696)
Supplement: Supporting Information — The Supporting Information are about WGCNA Scale independence and mean connectivity results. Supporting Figure S1. (A) Scale independence and mean connectivity results for IBD. (B) Scale independence and mean connectivity results for DR. The supporting informations have been uploaded. [file 9894696.f1.docx]

Supplementary Materials


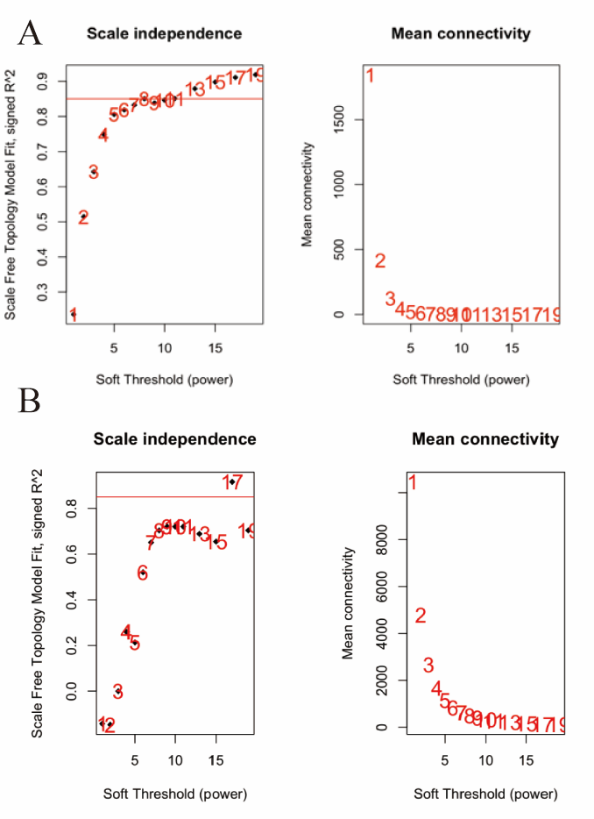


**Supplementary Figure 1**. WGCNA Scale independence and Mean connectivity results. (**A**) Scale independence and Mean connectivity results for IBD. (**B**) Scale independence and Mean connectivity results for DR.
